# Tuning Star Polymer Architecture to Tailor Secondary Structures and Mechanical Properties of Diblock Polypeptide Hydrogels for Direct Ink Writing

**DOI:** 10.1021/acs.biomac.4c01500

**Published:** 2024-12-19

**Authors:** Muireann Cosgrave, Kulwinder Kaur, Christopher Simpson, Łukasz Mielańczyk, Ciara Murphy, Robert D. Murphy, Andreas Heise

**Affiliations:** † Department of Chemistry, RCSI University of Medicine and Health Sciences, Dublin D02 YN77, Ireland; ‡ The SFI Centre for Advanced Materials and BioEngineering Research, RCSI University of Medicine and Health Sciences, Dublin D02 YN77, Ireland; § School of Pharmacy and Biomolecular Sciences, RCSI University of Medicine and Health Sciences, Dublin D02 YN77, Ireland; ∥ Tissue Engineering Research Group, Department of Anatomy and Regenerative Medicine, RCSI University of Medicine and Health Sciences, Dublin D02 YN77, Ireland; ⊥ Department of Histology and Cell Pathology, Faculty of Medical Sciences in Zabrze, Medical University of Silesia, Katowice 40-055, Poland; # CÚRAM the SFI Research Centre for Medical Devices, Department of Chemistry, RCSI University of Medicine and Health Sciences, Dublin D02 YN77, Ireland; ¶ Trinity Centre for Biomedical Engineering, Trinity College Dublin, Dublin D02 R590, Ireland

## Abstract

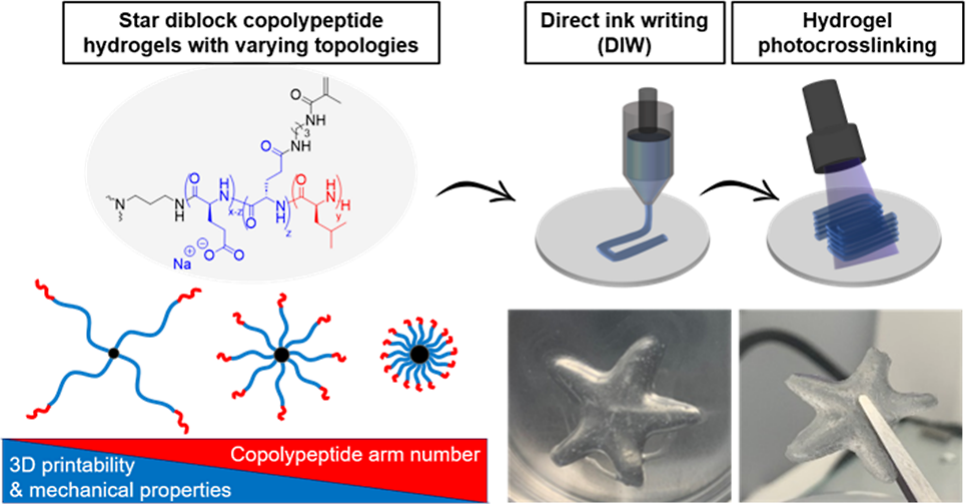

Hydrogel three-dimensional (3D) printing has emerged as a highly valuable fabrication tool for applications ranging from electronics and biomedicine. While conventional hydrogels such as gelatin, alginate, and hyaluronic acid satisfy biocompatibility requirements, they distinctly lack reproducibility in terms of mechanical properties and 3D printability. Aiming to offer a high-performance alternative, here we present a range of amphiphilic star-shaped diblock copolypeptides of l-glutamate and l-leucine residues with different topologies. Hydrophobic side chains of the l-leucine polymer block drive conformational self-assembly in water, spontaneously forming hydrogels with tunable mechanical properties, through variation of star topology. Their amenable shear-thinning and self-recovery properties render them suitable as hydrogel inks for direct ink writing. Well-defined 3D-printed structures can be readily generated and rapidly photo-cross-linked using visible light (405 nm) after methacrylamide functionalization, while hydrogel inks demonstrate good biocompatibility with top-seeded and encapsulated MC3T3 cells.

## Introduction

The field of 3D structural development has undergone significant progression in recent years, largely due to the integration of additive manufacturing, also known as 3D printing. Prior conventional methods for 3D fabrication, such as casting and electrospinning, have many limitations including long process times, low resolution, and lack of access to complex structures.^1^ 3D printing overcomes these obstacles with the assistance of computer aided design (CAD), enabling 3D part creation with higher complexity in shorter timeframes.^2−4^ Two of the most widely implemented techniques of printing are extrusion^5^ or light-based.^6,7^ Direct ink writing (DIW) is the most widely used extrusion technique, where materials of appropriate viscosity are continuously extruded into 3D structures via pneumatic, piston, or screw forces.^8^ For adequate printing resolution, key material design principles must be considered to provide shear-thinning and fast gelation properties.^9,10^ This is paramount with hydrogel filaments, which are ubiquitously used to generate 3D sensors, microfluidics and biomedical scaffolds,^9,11^ followed by enhancement of mechanical properties via conventional strategies such as photo-cross-linking.^12^ Through this, covalent chemical bonds are formed that preserve 3D structures for longer time periods than physical chemical bonds alone. Natural biopolymer hydrogels such as alginate,^13^ collagen,^14^ and gelatin^15^ are often employed due their ability to form physically bonded hydrogel networks, though they possess numerous limitations for 3D printing. They generally exhibit insufficient mechanical properties, a consequence of their undefined chemical structure and high batch-to-batch variability.^16,17^ Their mechanical strength can be augmented through chemical functionalization, where (meth)acryloyl chemistry is of high utility.^18^ These integrated functional groups can readily undergo free-radical polymerization (FRP) to form solid hydrogel networks with tunable mechanical properties.^19,20^ Despite this advancement, their undefined chemical structure means that accurate characterization of these biopolymer derivatives is a significant drawback. For example, gelatin methacryloyl (GelMA) characterization by ^1^H NMR spectroscopy is onerous due to the complex and ill-defined polypeptide structure.^21^

To this end, innovation is necessary to diversify and improve the library of 3D-printable hydrogels, with synthetic polypeptides ideally placed as highly regarded alternatives.^22,23^ They can be readily prepared on-scale by the ring opening polymerization (ROP) of the amino acid *N*-carboxyanhydride (NCA), of which a wide variety of amino monomers are now accessible due to recent advancements in synthetic protocols.^24^ NCA ROP can offer polypeptides with diverse polymer topologies, with high-control of chain composition and length.^25,26^ They are inherently biomimetic owing to their peptide backbone and synthetically adaptable to grant biocompatibility and modular mechanical properties, which can adequately simulate extracellular matrix (ECM).^27^ While our group has previously demonstrated the excellent 3D printability of these materials,^28−32^ the implementation of star topologies presents an opportunity to impart higher tuneability of polypeptide hydrogel systems.^33−35^

Herein, we explore a range of shear-thinning diblock copolypeptide hydrogels, consisting of l-glutamate (LGlu) and l-leucine (LLeu) building blocks, which have highly tunable mechanical properties based on varying the star architecture. The topological effects on the hydrogels’ mechanical properties were investigated through varying dendritic arm number while maintaining a constant monomer feed ratio of LGlu and LLeu residues. The materials spontaneously form physically cross-linked hydrogels through hydrophobic interactions within LLeu domains, endowing shear-thinning and self-recovery capabilities. Hydrogels could readily undergo DIW printing to form self-supporting 3D structures, which were then chemically fixated through photopolymerization of methacryloyl groups to vastly enhance mechanical properties of formed hydrogel networks, which had high cell viability in top-seeded and encapsulation assays.

## Experimental Section

### Materials

All chemicals were obtained from Sigma-Aldrich unless stated otherwise. *N*-(3-Aminopropyl)methacrylamide hydrochloride and amino acids were obtained from Fluorochem. Poly(propyleneimine) dendrimers generations 1–3 were obtained from SyMO-Chem.

### Methods

Nuclear magnetic resonance (^1^H NMR) analyses were completed using a Bruker Avance 400 (400 MHz) spectrometer at room temperature with CDCl_3_, DMSO, and trifluoroacetic acid-*d* (TFA-D). Attenuated total reflection FT-IR were recorded using a spectrometer (Thermo Scientific Nicolet iS10) in the region of 500–4000 cm^–1^. A background measurement was performed prior to the sample analysis. Eight scans were completed using a resolution of 2 cm^–1^. The molecular weight and molecular weight distributions (dispersity, *D̵* = *M*_w_/*M*_n_) were determined by gel permeation chromatography (GPC) in hexafluoroisopropanol (HFIP). Analyses were conducted on a PSS SECurity GPC system equipped with a PFG 7 μm 8 × 50 mm precolumn, a PSS 100 Å, 7 μm 8 × 300 mm, and a PSS 1000 Å, 7 μm 8 × 300 mm column in series and a differential refractive index detector at a flow rate of 1.0 mL min^–1^. The systems were calibrated against Agilent Easi-Vial linear poly(methyl methacrylate) (PMMA) standards and analyzed by the software package PSS winGPC UniChrom. Circular Dichroism (CD) spectroscopy was performed on an Applied Photophysics Chirascan Plus CD spectrometer at 20 °C. Polymer solutions were prepared a concentration of 0.01 mg/mL and analyzed in a quartz cuvette with a path length of 1 cm. Secondary structural features of recorded CD data were determined using online software BestSel, analyzing the region 195–250 nm. Rheological analyses were conducted on an MCR 102 digital rheometer (Anton Paar). All experiments were conducted at room temperature using a parallel plate (CP25–1, Anton Paar) consisting of a 25 mm diameter geometry and a gap length of 0.1 mm. The protective hood was used to avoid sample evaporation. For the photocuring study, the sample was analyzed immediately and irradiated with visible light (ThorLabs 510 mW visible lamp λ_max_ ∼ 405 nm) while on the rheometer plate 90 s into the measurement and was analyzed until a plateau was reached. The surface morphology was analyzed by scanning electron microscopy (SEM). Lyophilized cross-linked hydrogels were sliced to best visualize the internal structure of the network. Pore size analysis of each material was elucidated from the obtained images by manually measuring the pore size using ImageJ software. Fifty measurements were taken for each material, 4-SDC, 8-SDC, and 16-SDC. These were then imported into Stata for statistical analysis. The normality of the data was assessed prior to hypothesis testing and p values less than 0.05 were regarded as statistically significant.

### DIW 3D Printing

The hydrogel ink was formulated by the addition of 50 mg of polypeptide to 1 mL of 0.1 wt % lithium phenyl (2,4,6-trimethylbenzoyl) phosphinate (LAP) solution. The hydrogel was then mixed with 50 mg of comonomer (acrylamide or poly(ethylene glycol) methacrylate (PEGMA, *M*_n_ = 500 g/mol)). Initially, hydrogels were slowly injected into a syringe barrel using a 22 G needle to remove air bubbles. The syringe was then placed on the extrusion carriage of the 3D printer (discov3ry 3D, Structur3rd). Cura software (15.04.6) from Ultimaker was used to slice STL files used for DIW printing. After printing, the structures were then subjected to irradiation with visible light (ThorLabs 510 mW visible lamp λ_max_ ∼ 405 nm). Printing speeds were maintained at 25 mm/s for all 3D objects.

### In Vitro Biocompatibility

To determine the effect of 4-SDC hydrogel on cell activity, hydrogels were cultured with preosteoblast MC3T3-E1 cell line and monitored for cytotoxic indicators and proliferation. Cells were maintained in MEM alpha media (Biosera) supplemented with 10% fetal bovine serum (FBS), 1% glutamine, and 1% antibiotic and maintained at 37 °C in a humid environment with 5% CO_2_ in T175 flasks. At 80% confluence, cells were detached using trypsin–ethylenediaminetetraacetic acid (EDTA) treatment for 5 min at 37 °C. 17.5 × 10^5^ cells were seeded with the 4-SDC hydrogel in two ways: (i) mixed/encapsulated within the hydrogel and (ii) seeded directly on top of the hydrogel. Encapsulation was achieved by depositing 50 μL of hydrogel into each well, a thickness of ∼2.7 mm, and adding 5 μL volume of suspended cells (3 × 10^4^ cells) before mixing to ensure homogeneity. The volume of cell suspension in this case was kept to a minimum, so as not to dilute the final polymer concentration. Encapsulated hydrogels were then photo-cross-linked at 405 nm for 2 min to ensure chemical fixation. Top-seeded hydrogels were plated and cross-linked under the same conditions, before addition of 5 μL of cell suspension (3 × 10^4^ cells) directly onto the surface of the cross-linked hydrogels. A tissue culture grade coverslip with adhered cells was used as a positive control for comparison. All experimental cultures were conducted in 48-well tissue culture plates and supplemented with 200 μL of complete growth media. At day 1 postseeding, a lactate dehydrogenase (LDH) assay (CyQUANT LDH cytotoxic assay kit, Invitrogen) was carried out according to manufacturer’s instructions to determine cytotoxicity. In brief, 10 μL of 10X lysis buffer was added in one well of each sample and mixed by gentle tapping for maximum LDH activity. The well plate was incubated at 37 °C for 45 min following which 50 μL of each sample medium was transferred to a 96-well flat bottom in triplicate wells. 50 μL of the reaction mixture was then added to each well and mixed well. The plate was incubated at room temperature for 30 min protected from light. After 30 min, 50 μL of stop solution was added to each plate and mixed by gentle tapping. Absorbance was recorded at 490 and 680 nm using a plate reader. For double-stranded DNA (dsDNA) quantification, a Quant-iT PicoGreen assay kit (Invitrogen) was used. dsDNA quantification was assessed over a 7 day culture period to indirectly measure the proliferation of MC3T3-E1 cells on the prepared hydrogels according to manufacturer’s instructions; At 1, 3, and 7 days postseeding, media was removed and samples were rinsed with phosphate buffer saline (PBS) followed by addition of lysis buffer and stored at −80 °C. 3× freeze thaw cycles were performed to ensure complete cell and nuclear membrane lysis before running the assay. Lysates and dsDNA standard concentrations were plated with PicoGreen working solution in a black 96-well plate. Fluorescent emissions were read at 538 nm after excitation at 485 nm using a plate reader. Sample dsDNA quantities were calculated from the resultant linear regression equation of dsDNA standard concentrations vs fluorescent emission. The data are presented as the mean ± standard error of mean for the indicated number of separate experiments. Statistical analysis of data was performed with one-way analysis of variance and *p*-values less than 0.05 were considered significant.

### Synthesis of Star Diblock Copolypeptide (4 P(BLG_320_-*b*-LLeu_80_))

Benzyl-l-glutamate (BLG) NCA (5.00 g, 21.08 mmol) was dissolved in 75 mL of CHCl_3_ and 5 mL of DMF in a Schlenk flask at room temperature. G1 4-arm PPI dendrimer (22.16 mg, 0.07 mmol) was quickly added in one portion, and the flask was evacuated under vacuum to remove CO_2_. The reaction continued for 2 h, and a 1 mL aliquot was tested under FT-IR to confirm complete monomer consumption. Another aliquot was taken at this point for size exclusion chromatography (SEC) analysis. l-leucine NCA (828.29 mg, 5.27 mmol) in 40 mL of CHCl_3_ and 10 mL of DMF was quickly added to the reaction flask in one portion, and the reaction was allowed to continue until complete monomer consumption confirmed by FTIR spectroscopy (∼1.5 h). The copolymer was precipitated into excess diethyl ether and dried under vacuum to yield 4 P(BLG_320_-*b*-LLeu_80_) (yield: 4.195 g, 81%).

### Deprotection of Star Diblock Copolypeptide (4 P(LG_320_-*b*-LLeu_80_))

The diblock copolypeptide (4.195 g) was dissolved in 40 mL of trifluoroacetic acid in a flask. Once the polymer had solubilized, a six-times excess of HBr (33 wt % in acetic acid) was added dropwise to the solution. The reaction was capped with an outlet needle to vent HBr gas and allowed to stir for 18 h. The deprotected copolymer was precipitated into a ten times excess of diethyl ether using a centrifugation/decanting procedure. The polymer was then washed three times with diethyl ether and dried under vacuum in a desiccator. Following drying, the polymer was added to water and the pH was adjusted to 8/9 using NaOH. Once solubilized, the polymer was dialyzed against deionized water for 3 days and then lyophilized to yield 4 P(LG_320_-*b*-LLeu_80_) (yield: 1.537 g, 56%). *Protocol 2.2 and 2.3 were also used for 8 P(LG_320_-*b*-Lleu_80_) and 16 P(LG_320_-*b*-LLeu_80_) by replacing the G1 dendrimer with the G2 and G3, respectively.

### Synthesis of Methacrylamide-Functionalized Star Diblock Copolypeptide

The amphiphilic diblock copolypeptide (1.537 g, 0.03 mmol) was added to a one neck round-bottomed flask in 50 mL of deionized water. Targeting 20% functionalization of BLG units, *N*-(3-aminopropyl)methacrylamide hydrochloride (310.51 mg, 1.73 mmol) and 4-(4,6-Dimethoxy-1,3,5-triazin-2-yl)-4-methylmorpholium chloride (570.19 mg, 1.73 mmol) were added to the flask. The flask was wrapped in tinfoil, and the reaction was left under stirring for 2 days. The reaction mixture was precipitated into hydrochloric acid and washed three times with deionized water. It was then resuspended into NaOH and the pH was adjusted to 6. The copolymer was then dialyzed against deionized water for 3 days and then lyophilized to yield 4-SDC (yield: 950.60 mg, 62%).

## Results and Discussion

### Synthesis of Star Diblock Copolypeptide Hydrogels

**Scheme 1:**
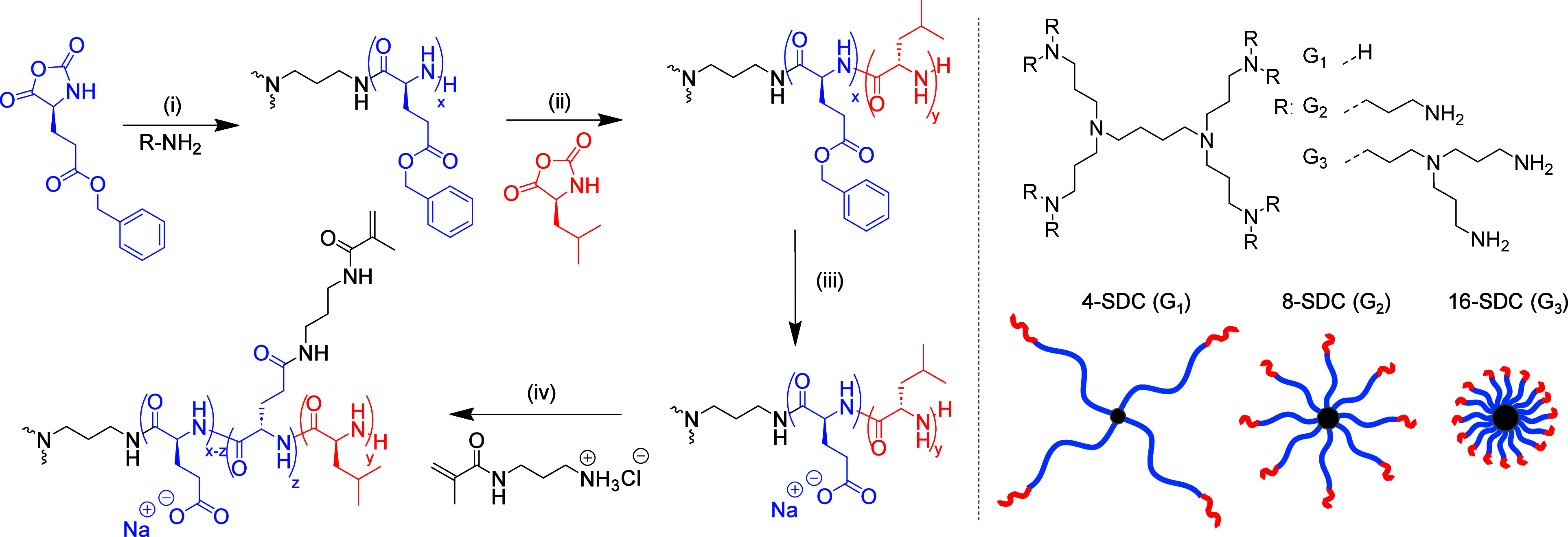
Synthetic Scheme of Star Diblock Copolypeptides from Poly(propylene imine) Dendrimers (4, 8, and 16 Arms) (i) CHCl_3_/DMF, 30 min, rt. (ii) CHCl_3_/DMF, 30 min, rt. (iii) HBr/TFA, overnight, rt; then NaOH. (iv) 3-(4,6-Dimethoxy-1,3,5-triazin-2-yl)-4-methylmorpholium chloride (DMTMM) and *N*-(3-aminopropyl)methacrylamide hydrochloride, H_2_O, 2 days, rt.

Prior to polymerization, l-leucine (LLeu) and benzyl-l-glutamate (BLG) *N*-carboxyanhydride (NCA) monomers were synthesized, as evidenced by ^1^H NMR and FTIR spectroscopy (Figures S1–S4). Star-shaped homopolypeptides of poly(benzyl-l-glutamate) (PBLG) were first synthesized via controlled ROP of BLG NCA, initiated by three generations of polypropyleneimine (PPI) dendrimers to yield star polymers of 4, 8, and 16 arms.^36−38^ Complete monomer conversion was confirmed by FTIR spectroscopy (Figure S6A). The star-shaped diblock copolypeptides were then obtained by in situ chain extension of PBLG with LLeu NCA, with FTIR spectroscopy (Figure S6B) confirming complete monomer conversion (Scheme 1). A monomer/initiator feed ratio of 320:80:1 (BLG NCA/LLeu NCA/NH_2_) was maintained for each of the star topologies, resulting in a constant total molecular weight and shorter diblock copolypeptide arm length with increasing PPI dendrimer generation. For example, the G1 PPI yields a polypeptide with 4 arms, with each arm comprising 80 units of BLG and 20 units of LLeu. Experimental monomer feed ratios were in good agreement with theoretical as confirmed by ^1^H NMR spectra (Figures S7, S10, S13), and diblock chain extension was confirmed by SEC analysis (Figures S8, S11, S14 and Table S1) with low dispersities (*D̵* < 1.2) observed. Removal of benzyl-protecting groups of BLG residues was completed by acid hydrolysis followed by NaOH neutralization yielding amphiphilic star diblock copolypeptides. The sodium l-glutamate (LGlu) residues were then functionalized via postpolymerization modification with *N*-(3-aminopropyl)methacrylamide (target ∼20% conversion of glutamic acid units) in the presence of 4-(4,6-dimethoxy-1,3,5-triazin-2-yl)-4-methyl-morpholinium chloride (DMTMM), to yield 4, 8, and 16 arm star diblock copolypeptides (SDCs) termed 4-SDC, 8-SDC, and 16-SDC, respectively. The degree of functionalization of each of the star copolypeptides (4-SDC, 8-SDC, and 16-SDC) was in good agreement (54, 65, 70 of 320) with the targeted value (64 of 320), as confirmed by ^1^H NMR spectra (Figures S15–S17).

### Influence of Star Topology on Secondary Structure and Rheological Properties

**Figure 1:**
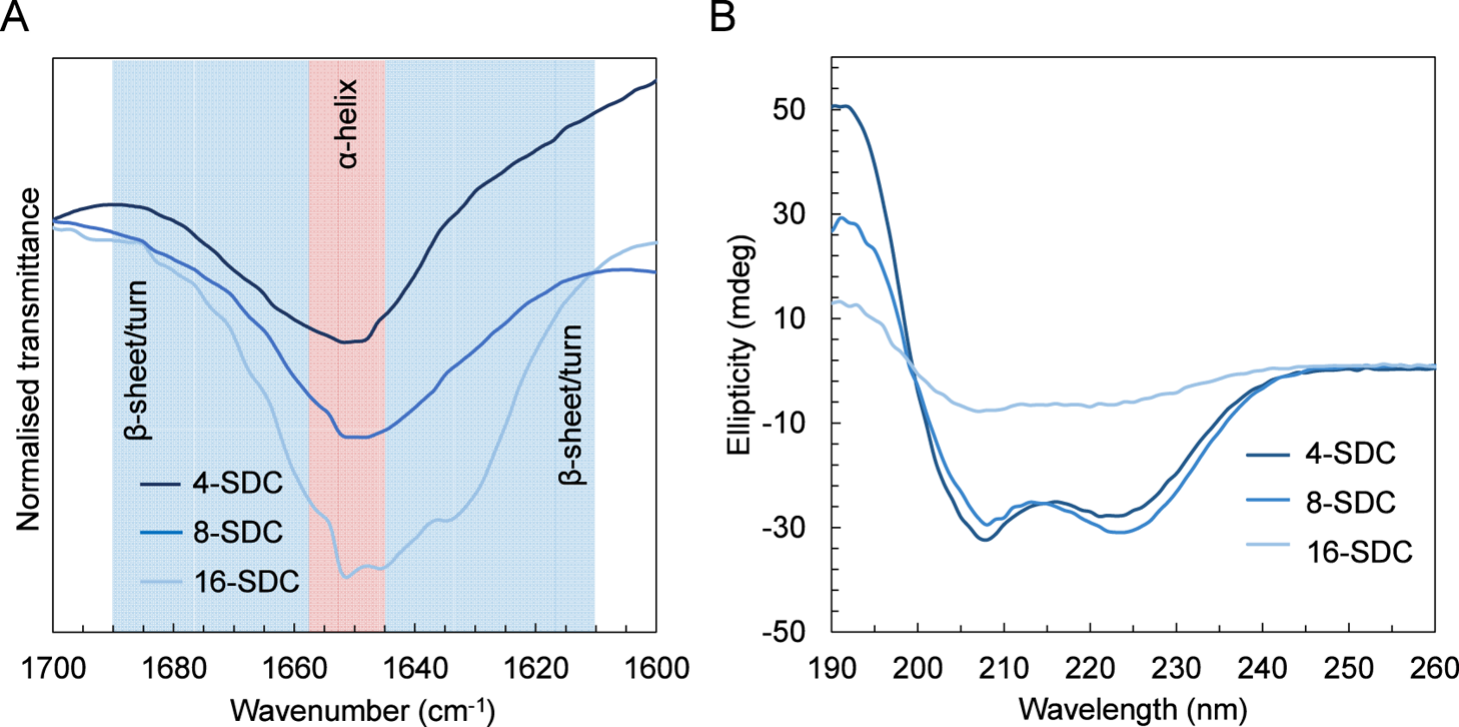
Secondary structure analysis of 4-SDC, 8-SDC, and 16-SDC by FTIR (A) and CD spectroscopy (B).

The secondary structural regimes and amide I bands for the SDCs were determined by using ATR-FTIR and CD spectroscopy (Figure 1). For all samples, predominant α-helical bands between 1648 and 1654 cm^–1^ were observed (Figure 1A). While small bands at 1645 and 1634 cm^–1^ might suggest random coil or β-motifs for 16-SDC, this cannot be unambiguously concluded without further analysis. CD spectra corroborated the secondary structure trend observed in the FTIR studies (Figure 1B). CD spectra of 4-SDC and 8-SDC display distinct patterns of α-helical structures with two minima at 208 and 225 nm, both exhibiting ∼73% α-helix content. These bands are significantly weaker for 16-SDC, with a value of ∼21% suggesting a much reduced extent of secondary structures. It is suspected that this decrease in conformational order with increasing arm number is likely due to shorter polypeptide arm lengths, which has been previously observed by the Deming group for linear block copolypeptides.^39^ Shorter l-glu arm sequences may also undertake mixed conformations due to their connectivity with terminal hydrophobic l-leu chains, as observed from the collated CD results in Table S2.

**Figure 2:**
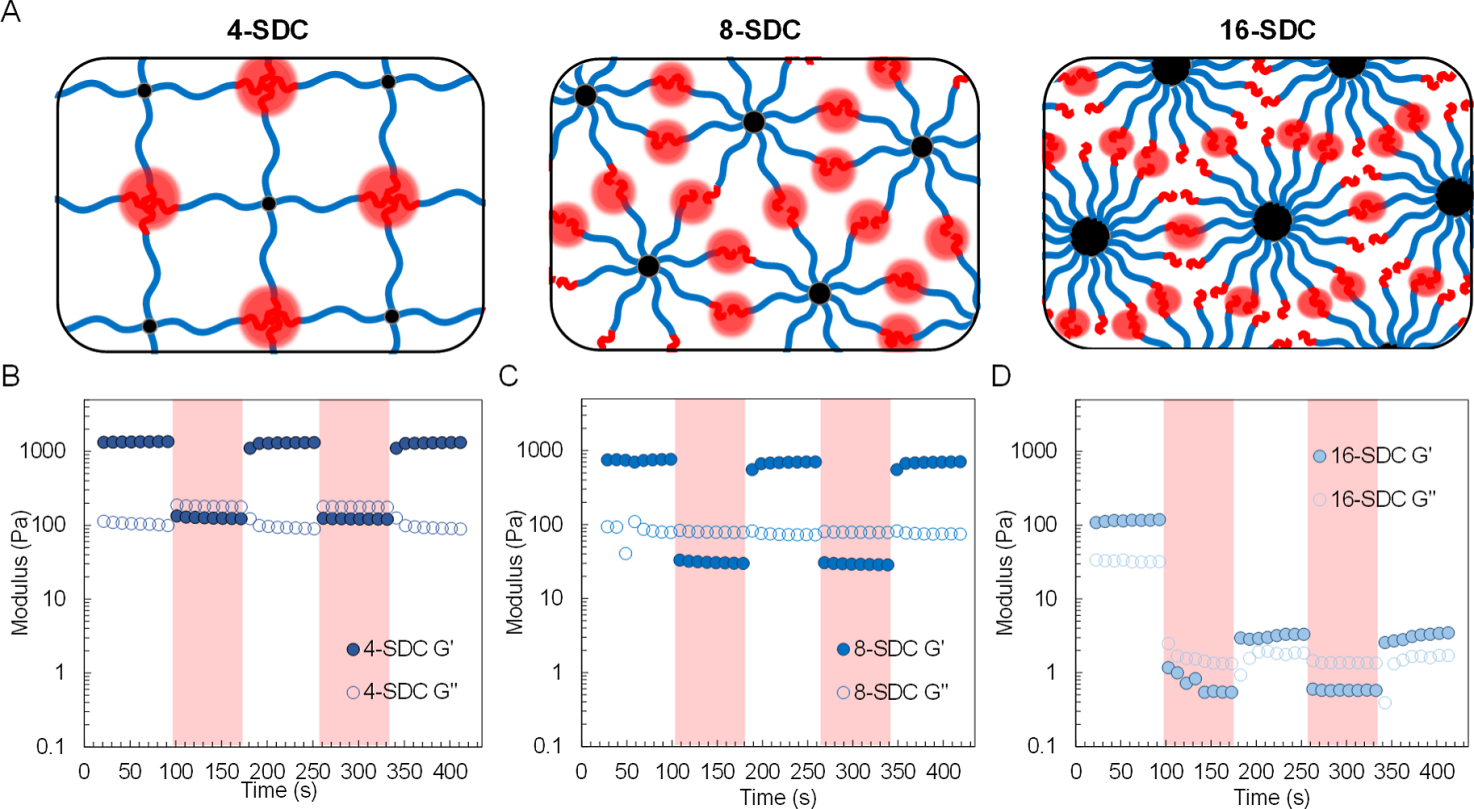
Graphical representation (A) of different physically assembled SDC hydrogel networks. Rheological analysis of 4-SDC (B), 8-SDC (C), and 16-SDC (D) hydrogels using dynamic amplitude sweep at 5.0 wt % (γ = 0.1% for 240 s (white), then γ = 40% for 240 s (red), then γ = 0.1% for 240 s, then γ = 40% for 240 s, then γ = 0.1% for 240 s, all at ω = 1 rad/s).

Rheological studies of the self-assembled SDC hydrogels (Figure 2A) were then conducted to identify shear regimes and mechanical property changes for different topologies (4-SDC, 8-SDC, and 16-SDC). SDC hydrogels were formulated at 5.0 wt % in the presence of 0.1 wt % LAP photoinitiator and 5.0 wt % PEGMA (*M*_n_ 500 g/mol) as a comonomer. A dynamic amplitude sweep was employed to determine the shear-thinning and self-recovery abilities of the hydrogels, where steps in strain were varied between low strain (γ = 0.1%) and high strain (γ = 40%; Figure 2B–D).

Polymer topology appeared to affect the recovery of the hydrogel physical networks following shear strain application. Both 4-SDC and 8-SDC hydrogels fulfilled each of the predetermined parameters, recovering rapidly to the original storage moduli (*G*′) after high strain (40%). Specifically, the initial storage moduli of 4-SDC (1330 Pa) and 8-SDC (760 Pa) recovered almost instantaneously to 1322 and 704 Pa, respectively, after return to low strain (0.1%), satisfying desirable properties for DIW printability. In contrast, the 16-SDC hydrogel exhibited poor self-recovery properties. The initial storage modulus of 120 Pa diminished to 3 Pa following application and removal of high strain (40%), It is possible that the shorter LLeu chain length (5) per arm are “crowded out” by the higher number of copolypeptide arms (16), limiting the mobility of the arms to reassemble to the original network. This is anticipated to result in diminished mechanical properties for 16-SDC, which excluded the hydrogel from consideration for the DIW.

**Figure 3:**
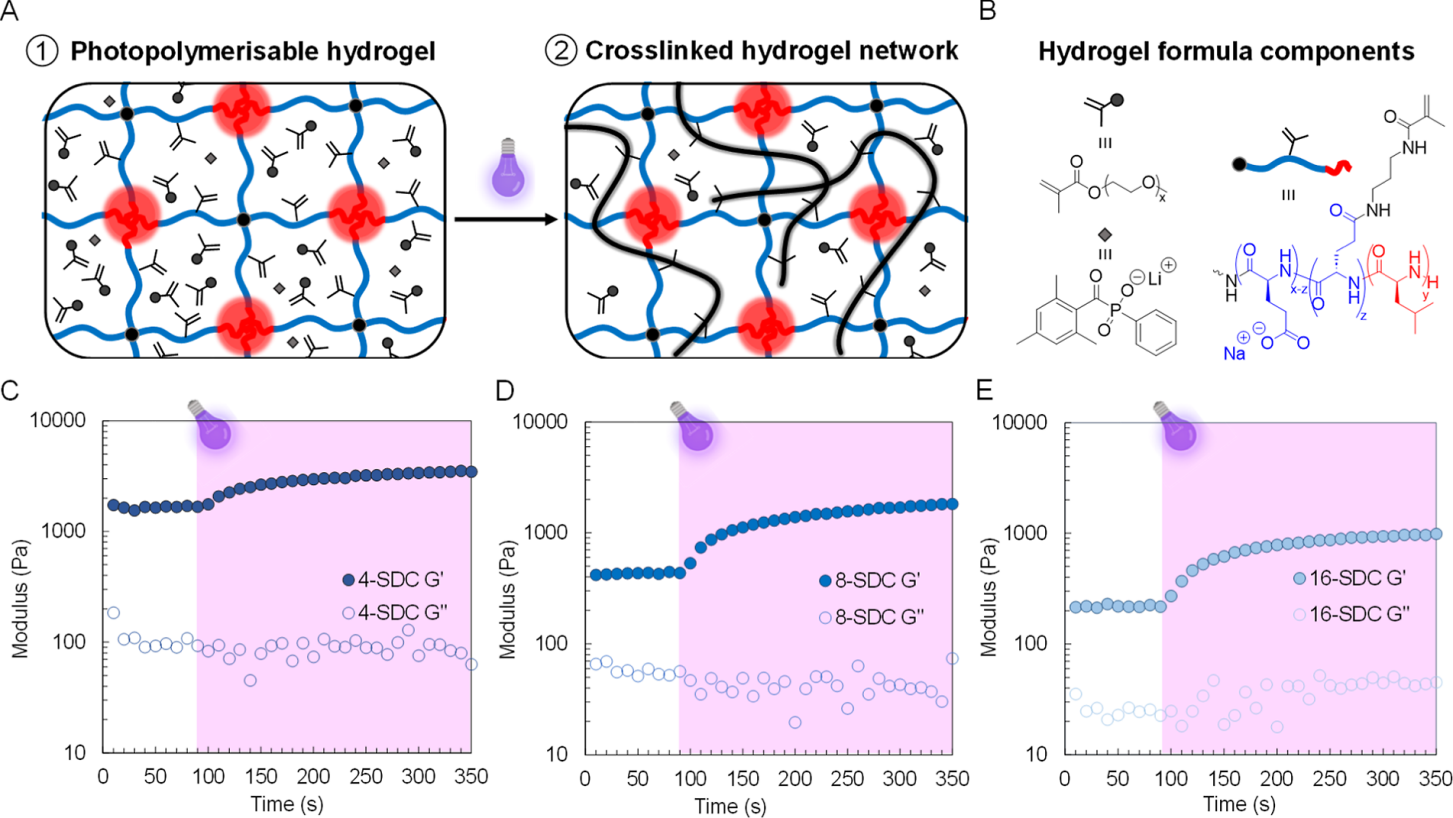
Physical hydrogel network (A1) undergoes photoirradiation at 405 nm to introduce a covalent network (A2). Hydrogel formulation comprised 5.0 wt % of the respective SDC in 0.1 wt % LAP solution and 5.0 wt % PEGMA comonomer (B). Rheological time curing sweep, showing photo-cross-linking of hydrogel comprising 5.0 wt % 4-SDC (C), 8-SDC (D), and 16-SDC (E). Irradiation began at 90 s and was continuous using 405 nm LED (6 mW/cm^2^); all experiments were performed at γ = 0.1% and ω = 1 rad/s.

The photo-cross-linking regime of the hydrogels was then profiled by a rheological time curing sweep to investigate changes in storage modulus upon photoirradiation (Figure 3 and Table 1). SDC hydrogel formulations were maintained at 5.0 wt % with 0.1 wt % LAP and 5.0 wt % PEGMA (Figure 3B). After a 90 s incubation time, irradiation at 405 nm (6 mW/cm^2^) led to a rapid increase in the storage modulus for all SDC hydrogels, indicative of chemical cross-links formed by FRP networks (Figure 3C–D). The cross-linking profile was similar for all SDCs, with a plateaued storage modulus observed almost 200 s after irradiation. 4-SDC displayed the strongest mechanical properties, with a storage modulus of 3480 Pa, while moduli of 1830 and 990 Pa were noted for 8-SDC and 16-SDC, respectively. Acrylamide as a comonomer was also trialed to identify differences in photo-cross-linking regimes. Slightly faster photo-cross-linking kinetics were observed (Figure S18), while all hydrogels demonstrated higher storage moduli than those with PEGMA, with 8560, 26740, and 4440 Pa observed for 4-SDC, 8-SDC, and 16-SDC, respectively. Despite acrylamide’s mechanical performance advantages and while polyacrylamide is a commonly used biomedical hydrogel,^40^ the monomer is known to be cytotoxic.^41^ Therefore, PEGMA was selected for subsequent experiments due to its biocompatibility^42^ while also offering favorable photo-cross-linking kinetics and mechanical properties.

**Table 1:** Hydrogel Properties of Star Diblock Copolypeptide Series

polymer^a^	*M*_n_^b^(g/mol)	modulus (Pa)^c^	modulus (Pa)^d^	mesh size (μm)^e^
4-SDC	58,000	1330	3480	0.17 ± 0.07
8-SDC	58,500	760	1830	0.19 ± 0.07
16-SDC	59,500	120	990	0.24 ± 0.13

a Polymer used for photo-cross-linkable hydrogel resin formed at 5 wt % polymer, 5 wt % comonomer (PEGMA), and 0.1 wt % photoinitiator (LAP).

b Molecular weight as determined by^1^H NMR spectroscopy.

c Initial storage modulus (*G*′) of hydrogel ink prior to photo-cross-linking.

d Plateaued storage modulus (*G*′) of photo-cross-linked hydrogel.

e Calculated using ImageJ from SEM images.

Hydrogel surface morphology was also evaluated via SEM to probe the microarchitectural organization of polymer networks.^43^ Identical photo-cross-linked hydrogels of the same composition as in rheological tests were prepared and lyophilized to determine variance in morphology and pore sizes due to star topology (Figure S19). SEM images confirmed the anticipated variations in surface morphology and porosity of relevance for cell proliferation^44^ and media diffusion. ImageJ was used to obtain readings on average pore size of the lyophilized hydrogels, followed by Stata input for statistical analysis. Pore sizes increased with increasing polymer arms, with 4-SDC averaging 0.17 ± 0.07 μm, increasing to 0.19 ± 0.07 μm and 0.24 ± 0.13 μm for 8-SDC and 16-SDC, respectively. The statistical significance of this trend was evaluated by implementing a Kruskal–Wallis test in Stata. This yielded a p-value less than 0.05 (0.002), signifying statistical significance and confirming the impact of polymer topology on surface morphology features. The ability to control such features lends great potential to SDCs as alternative, more tunable hydrogel inks than conventional biopolymer hydrogels.

### DIW 3D Printing and Biocompatibility

**Figure 4:**
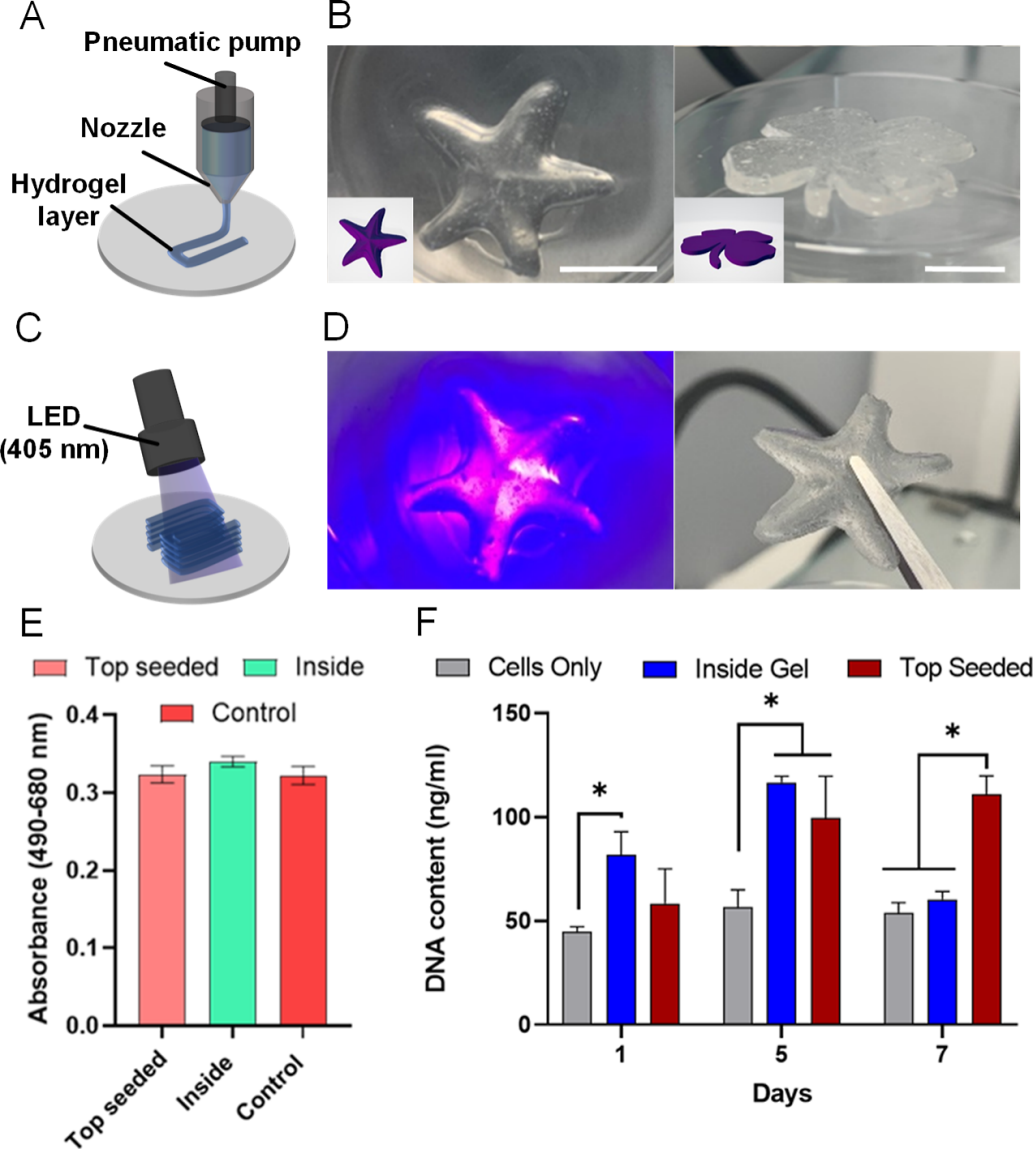
DIW printing (A) and 3D-printed structures (B) using 4-SDC hydrogel formulation compared to the corresponding CAD files (scale bar: 1 cm). Post DIW photo-cross-linking (C) of hydrogel structures to enhance mechanical properties (D). LDH assay (day 1) of 4-SDC with MC3T3 cell line (E). DNA content (F) within the hydrogels as a result of different seeding techniques (D) *p < 0.05. Cells were seeded on top and encapsulated within the hydrogel for both assays.

The 4-SDC hydrogel was identified as a lead candidate from rheological analysis and was further explored for the DIW 3D printing. The hydrogel could print a continuous filament with minimal optimization using a 22-gauge (G) needle (Figure 4A), with high structural integrity and could render high-fidelity 3D structures such as a starfish or shamrock, resembling the input CAD files (Figure 4B). Once printed, the formed 3D structures were fixed by photo-cross-linking at 405 nm (Figure 4C), forming an elastic solid material (Figure 4D) from the original soft, dispersible hydrogel.

The cytotoxicity of the 4-SDC hydrogel was then investigated using a LDH assay (Figure 4E), where LDH is a cytosolic enzyme that released when the cell membrane is ruptured or has lost integrity and is a widely used indicator of in vitro cytotoxicity.^45,46^ The assay revealed negligible cytotoxicity to MC3T3 cells, an osteoblast cell line, which were encapsulated within and seeded on the top of the hydrogel after day 1. This indicates a high potential for maintaining cell viability during the encapsulation of preosteoblasts. dsDNA content was then assessed across the formulated hydrogel range over the 7 day culture period to determine levels of cell attachment and proliferation (Figure 4F). A greater amount of dsDNA content was observed in encapsulated cell hydrogels compared to control at day 1, indicating a high amount of retained, viable cells inside the hydrogel, where there was no significant difference on day 1 between the top-seeded group and the control, further validating the biocompatibility of the 4-SDC hydrogel. Beyond this, at day 5 dsDNA content was further increased over controls, showing significance over control in both the encapsulation gel and top-seeded groups, indicating enhanced cell proliferation. The increased level of dsDNA content was maintained at day 7 for the top-seeded group, while in the encapsulation gel group, the dsDNA content decreased to levels comparable to the control. It is hypothesized that hydrogels cannot maintain increased levels of proliferation for encapsulated cells upon reaching maximum confluency, resulting in reduced dsDNA content.^47^ Nonetheless, the inside gel dsDNA content is comparable to the control, inferring that the hydrogel is cell-adhesive and may provide a favorable microenvironment for osteoblast proliferation and survival. For the top-seeded group, the hydrogel demonstrates the ability to enhance osteoblast proliferation compared to the control, indicative of its potential to support bone cell growth. Thus, the combined bioactivity, biocompatibility, and microstructure of the 4-SDC hydrogel may contribute to the improved cell proliferation observed.

## Conclusions

A series of hydrogels with tailorable properties were developed from amphiphilic diblock copolypeptides of varying star architectures and fixed molecular weights. Hydrogel mechanical properties, rheological regimes, and network pore sizes were directly influenced by star topology through variation in secondary structure conformations. Postpolymerization modification with methacryloyl groups provided hydrogels with functionalities for photo-cross-linking using visible light (405 nm), which enabled rapid mechanical property changes. Of the series, the 4-armed star diblock copolypeptide demonstrated highly desirable rheological properties for subsequent DIW, producing high-fidelity 3D structures. The hydrogel could also support preosteoblast attachment in both top-seeded and encapsulated environments and has great potential to support cell proliferation when encapsulated. The high DIW printability and cytocompatibility of the hydrogel material may offer a viable alternative for future biomedical applications, broadening the scope of the 3D-printing hydrogel library and helping to bridge the gap between synthetic and natural materials.

## Supporting Information Available

Synthesis and characterization of NCAs, NMR, GPC, and FTIR analysis of polypeptides, rheology plots, and SEM images (PDF)
