# Supplementary material for: Tuning Star Polymer Architecture to Tailor Secondary Structures and Mechanical Properties of Diblock Polypeptide Hydrogels for Direct Ink Writing
Source: Biomacromolecules. 2024 Dec 19;26(1):670–8. doi: 10.1021/acs.biomac.4c01500 (PMC11733935; doi:10.1021/acs.biomac.4c01500)
Supplement: Supplementary file 1 — bm4c01500_si_001.pdf [file bm4c01500_si_001.pdf]

Supporting Information

Tuning Star Polymer Architecture to Tailor Secondary Structures and Mechanical Properties of Diblock Polypeptide Hydrogels for Direct Ink Writing

Muireann Cosgrave,[1,2] Kulwinder Kaur,[3,4] Christopher R Simpson,[3,4] Łukasz Mielańczyk,[5] Ciara Murphy,[2,4,7] Robert D Murphy*,[1] Andreas Heise*,[1,2,7]

1 Department of Chemistry, RCSI University of Medicine and Health Sciences, Dublin, D02 YN77, Ireland

2 The SFI Centre for Advanced Materials and BioEngineering Research, RCSI University of Medicine and Health Sciences, Dublin, D02 YN77, Ireland

3 School of Pharmacy and Biomolecular Sciences, RCSI University of Medicine and Health Sciences, Dublin, D02 YN77, Ireland

4 Tissue Engineering Research Group, Department of Anatomy and Regenerative Medicine, RCSI University of Medicine and Health Sciences, Dublin, D02 YN77, Ireland

5 Department of Histology and Cell Pathology, Faculty of Medical Sciences in Zabrze, Medical University of Silesia, Katowice, 40-055, Poland.

6 CÚRAM the SFI Research Centre for Medical Devices, Department of Chemistry, RCSI University of Medicine and Health Sciences, Dublin, D02 YN77, Ireland

7 Trinity Centre for Biomedical Engineering, Trinity College Dublin, Dublin, D02 R590, Ireland

1. Experimental

2. Synthetic protocols

2.1 Synthesis of benzyl-L-glutamate *N*-carboxyanhydride (BLG NCA). Triphosgene (3.13 g, 10.53 mmol) and epichlorohydrin (7.80 g, 84.30 mmol) were initially dissolved in 130 mL of THF. To this, y-benzyl-L-glutamate (5.00 g, 21.07 mmol) was added in one portion and the reaction suspension was heated under reflux. The reaction continued until all solids dissolved and the solution became clear (~ 1-2 hours). The solution was then cooled under argon, filtered and reduced to one third of its original volume with the use of a cold trap. The NCA was precipitated by addition of 200 mL of hexane and it was stored overnight at -18 °C. The solid NCA was dried under vacuum and recrystallized from ethyl acetate. This solution was precipitated into excess hexane and filtered (2-3 times). It was then vacuum dried to yield a white fluffy solid (yield: 4.50 g, 81.5%). *The same protocol was applied for the synthesis of L-Leucine (L-Leu) NCA synthesis (yield: 2.40 g, 40%).

3. Additional Figures

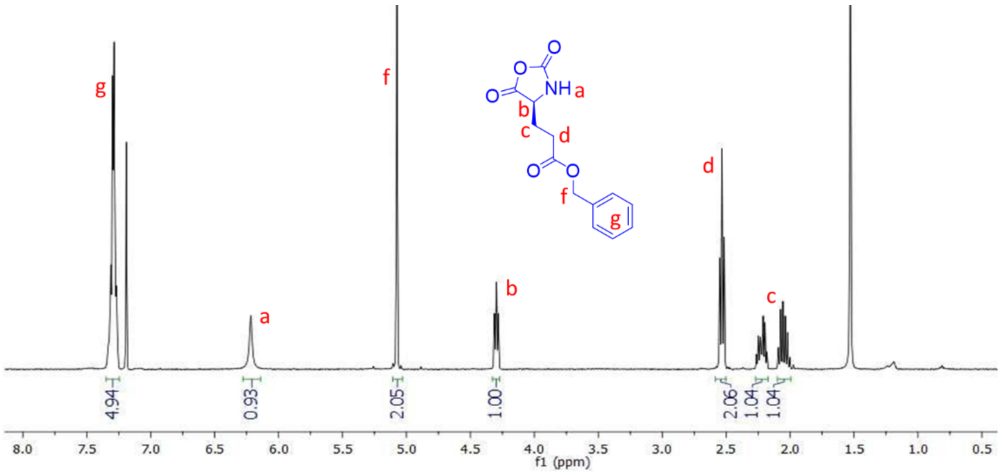

Figure S1. ^{1}H NMR spectra of BLG NCA in $CDCl_3$.

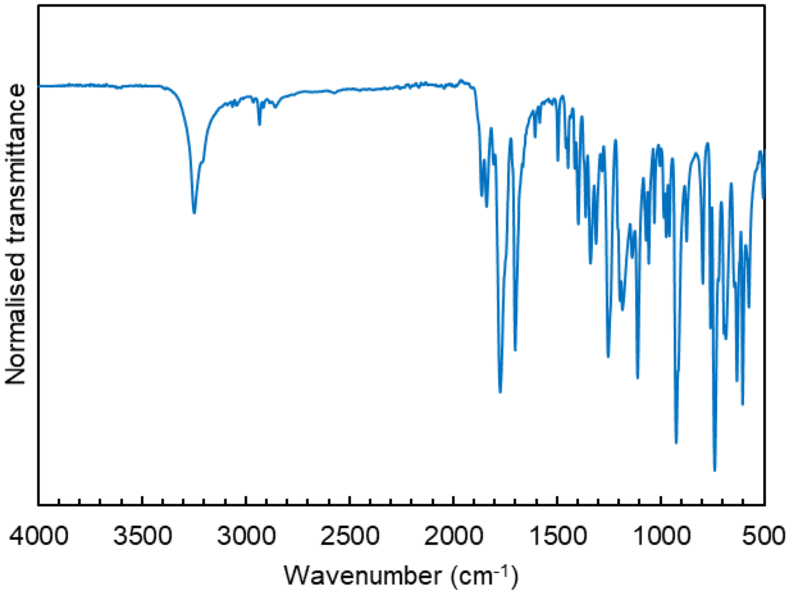

Figure S2. FTIR spectra of BLG NCA.

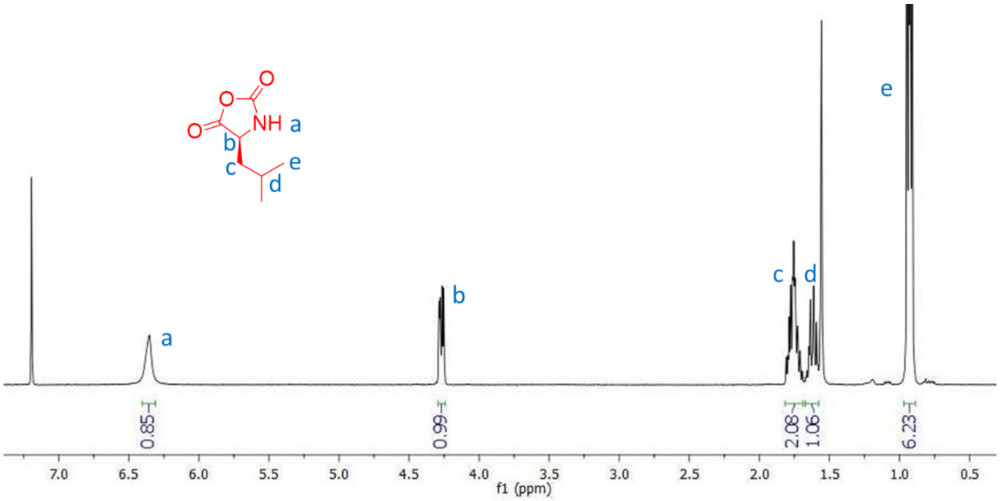

Figure S3. ^{1}H NMR spectra of L-Leu NCA in $CDCl_3$.

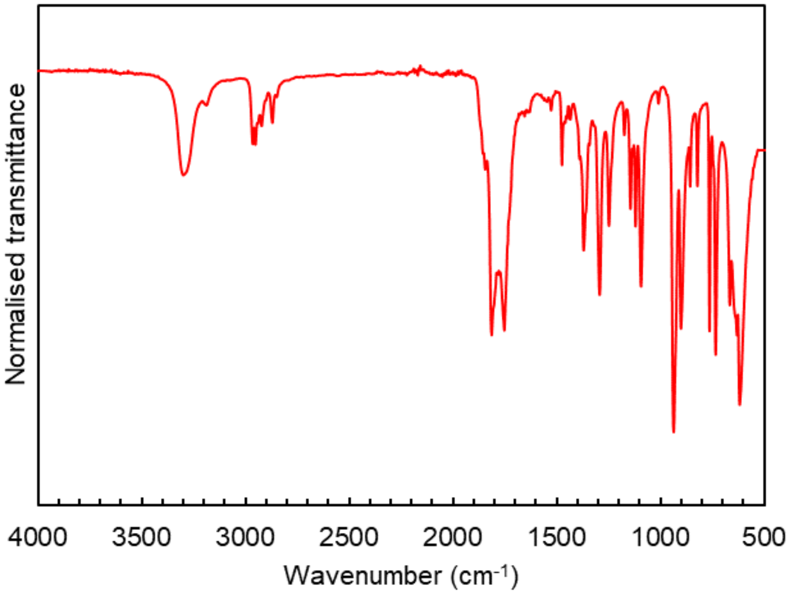

Figure S4. FTIR spectra of L-Leu NCA.

Table S1. Molecular weight and dispersity characteristics of star polypeptides.

**Polymer**	$M_n{}^a$ **(g/mol)**	$M_n{}^b$ **(g/mol)**	$M_n{}^c$ **(g/mol)**	$Đ_M{}^c$
4-P(BLG_{320})	70,800	70,800	56,000	1.28
4-P(BLG_{320}-*b*-$LLeu_{80}$)	83,000	83,500	69,700	1.21
8-P(BLG_{320})	71,300	71,300	32,600	1.20
8-P(BLG_{320}-*b*-$LLeu_{80}$)	83,500	85,000	44,000	1.17
16-P(BLG_{320})	72,200	72,200	49,200	1.19
16-P(BLG_{320}-*b*-$LLeu_{80}$)	84,400	85,000	58,200	1.11

[a]Theoretical molecular weight based on stoichiometric feed ratios. [b]Molecular weight as determined by ^{1}H-NMR spectroscopy. [c]Molecular weight and dispersities determined by SEC (PMMA standards).

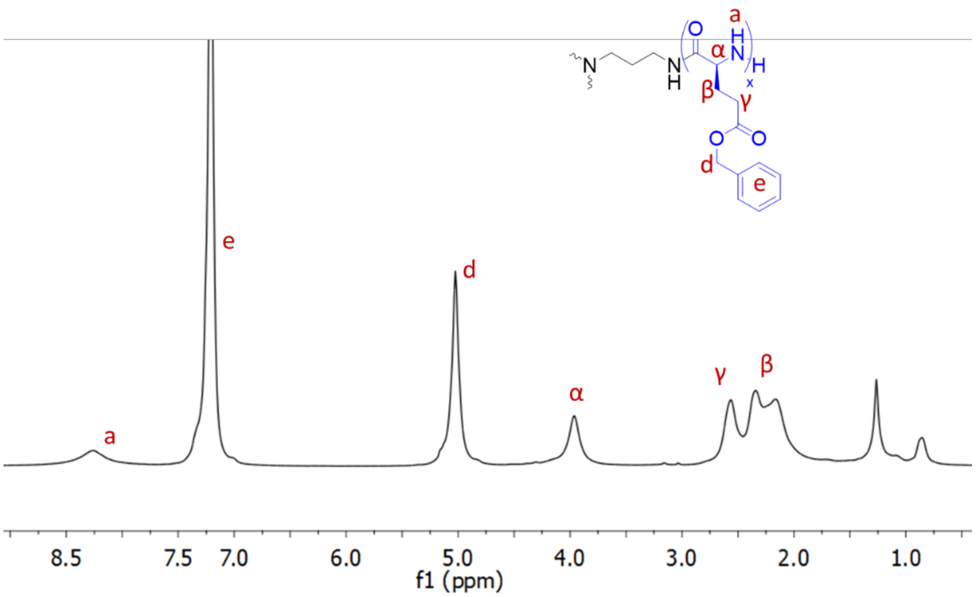

Figure S5. ^{1}H NMR spectra of 4-P(BLG_{320}) in $CDCl_3$.

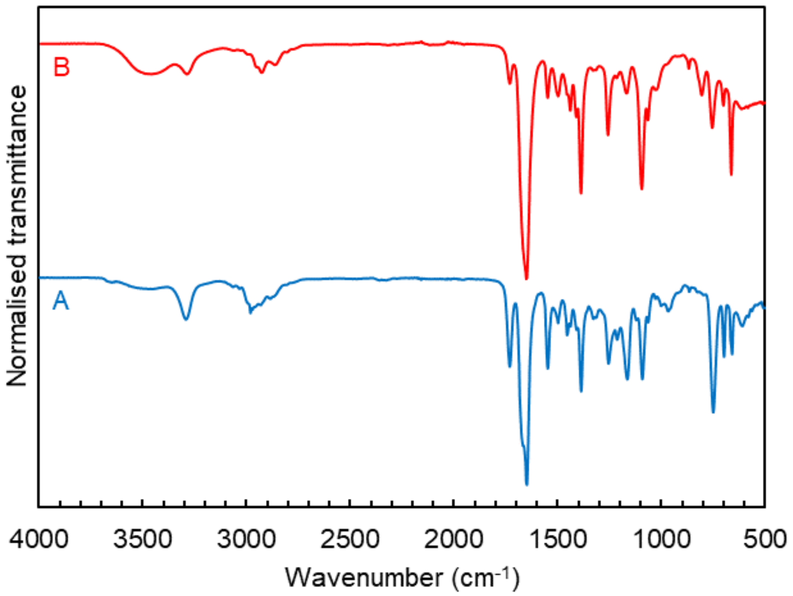

Figure S6. FTIR spectra of (A) 4-P(BLG_{320}) and (B) 4-P(BLG_{320}-*b*-$LLeu_{80}$).

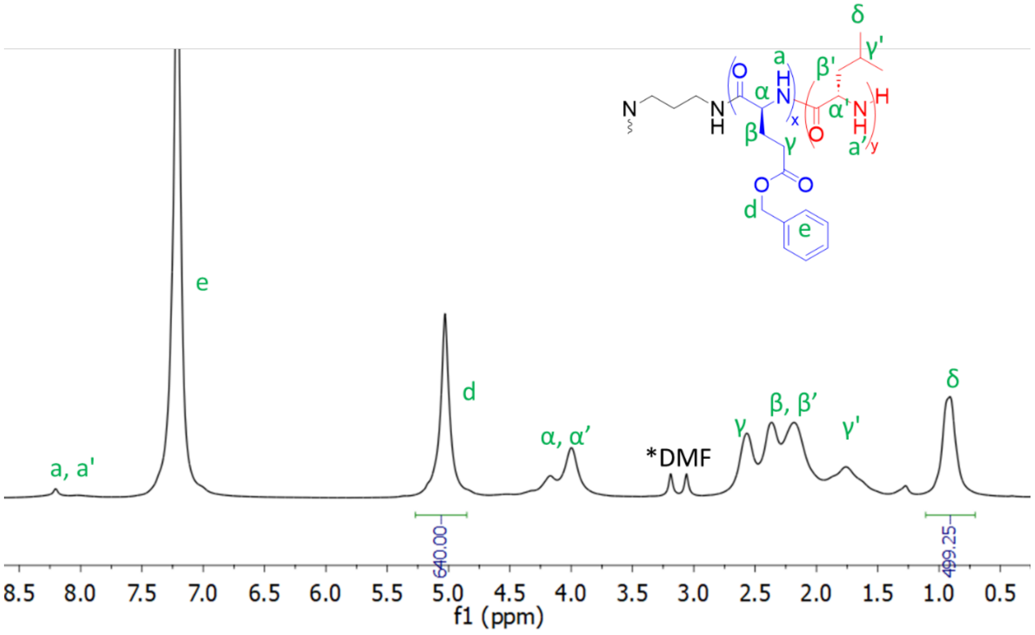

Figure S7. ^{1}H NMR spectra of 4-P(BLG$_{320}$-*b*-LLeu$_{80}$) in $CDCl_3$.

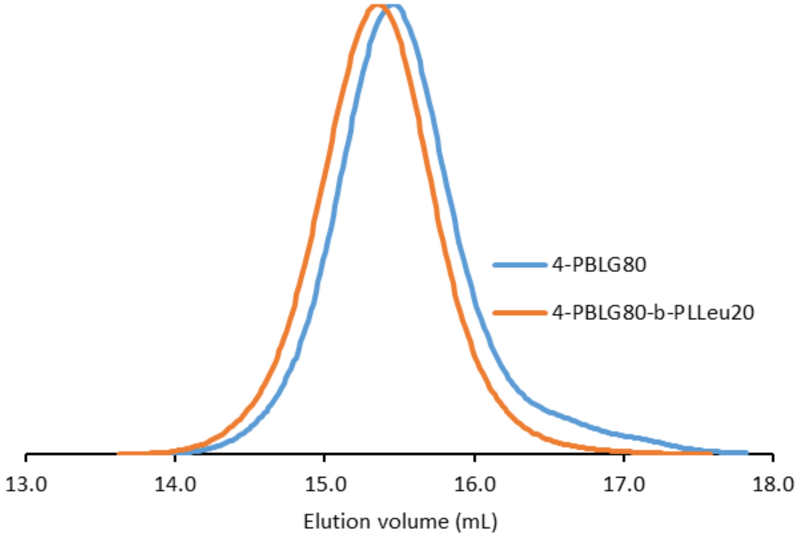

Figure S8. GPC trace illustrating peptide chain extension of 4-P(BLG$_{320}$) to 4-P(BLG$_{320}$-*b*-LLeu$_{80}$).

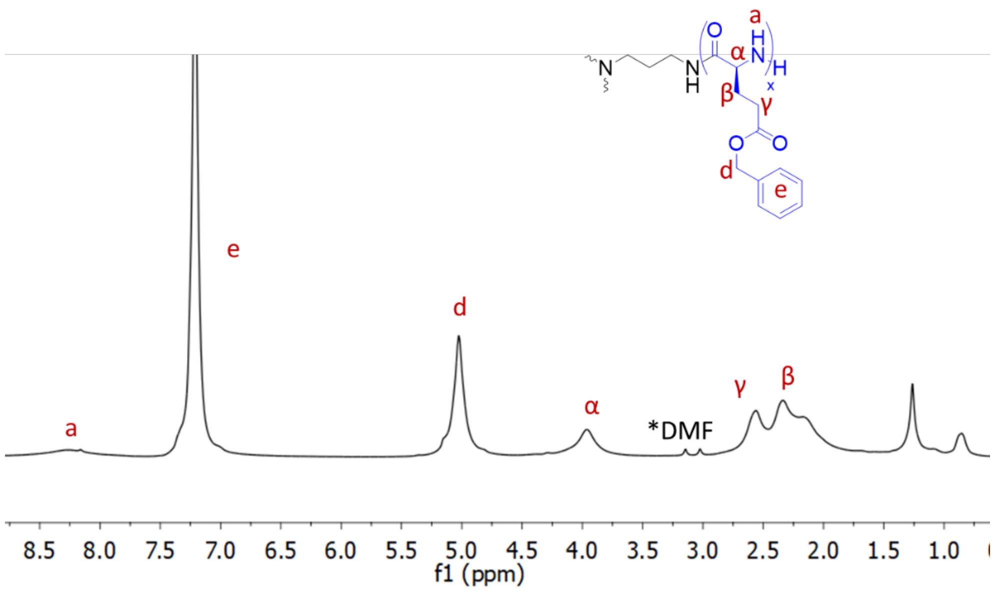

Figure S9. ^{1}H NMR spectra of 8-P(BLG$_{320}$) in $CDCl_3$.

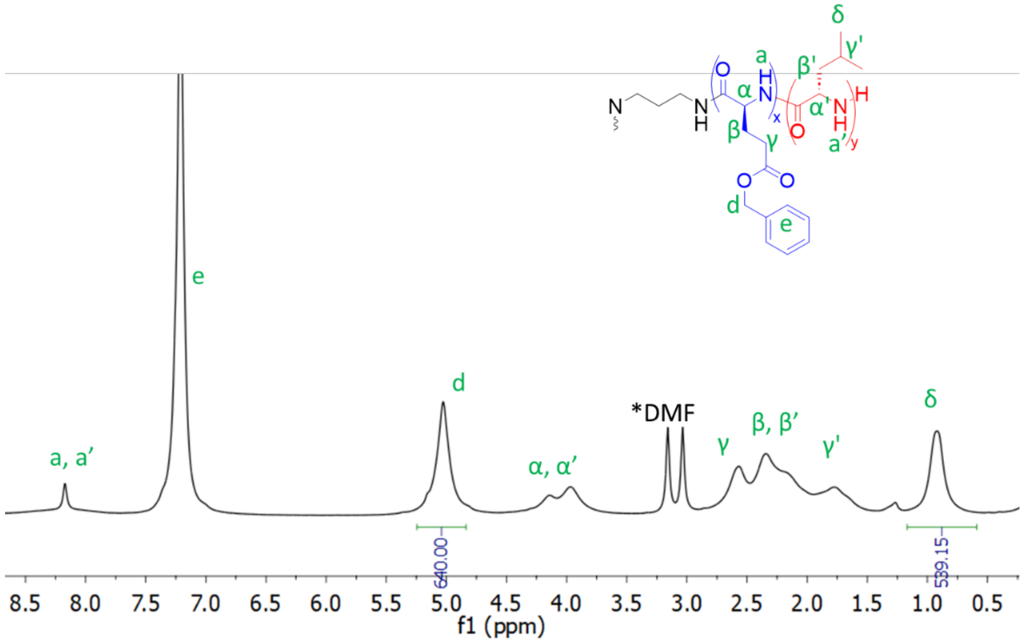

Figure S10. ^{1}H NMR spectra of 8-P(BLG$_{320}$-*b*-LLeu$_{80}$) in $CDCl_3$.

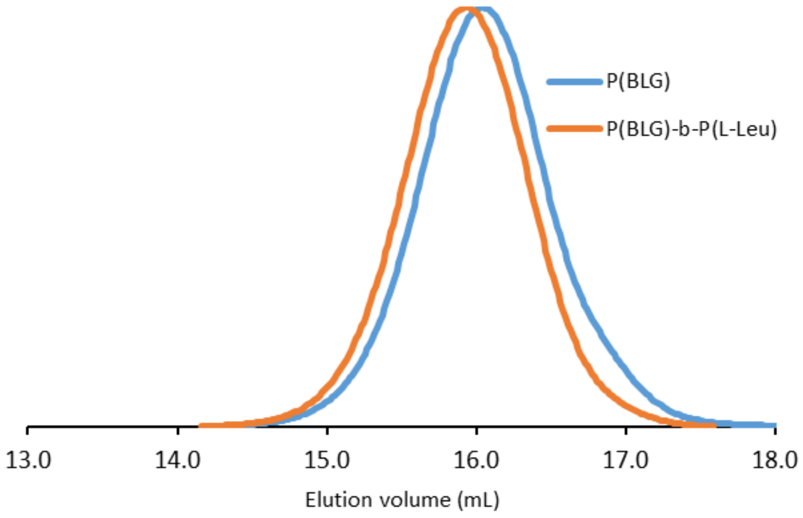

Figure S11. GPC trace illustrating peptide chain extension of 8-P(BLG_{320}) to 8-P(BLG_{320}-*b*-$LLeu_{80}$).

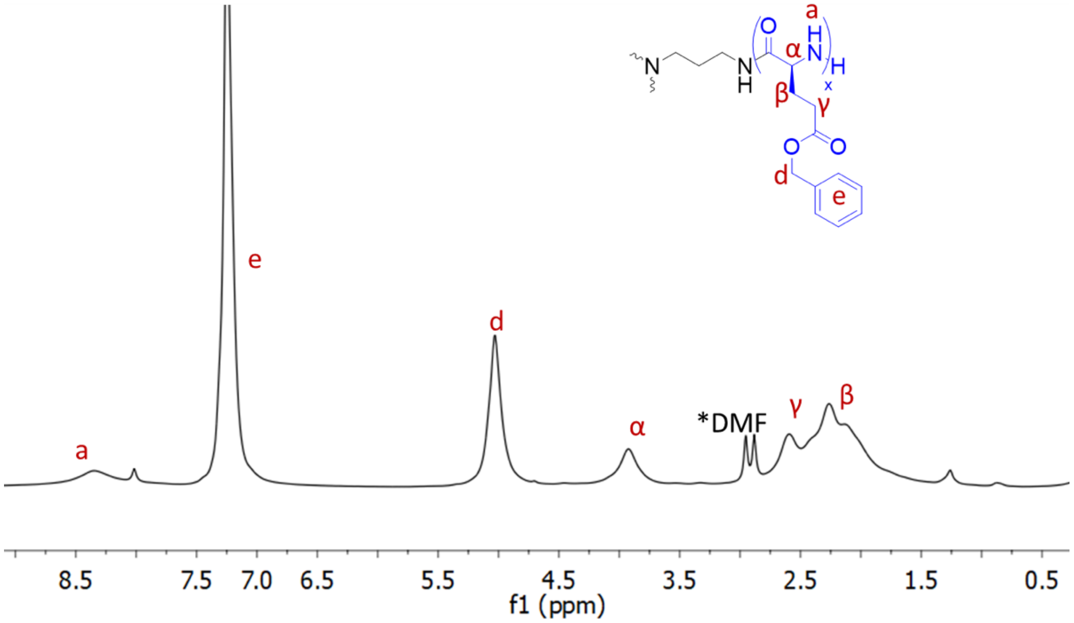

Figure S12. ^{1}H NMR spectra of 16-P(BLG_{320}) in $CDCl_3$.

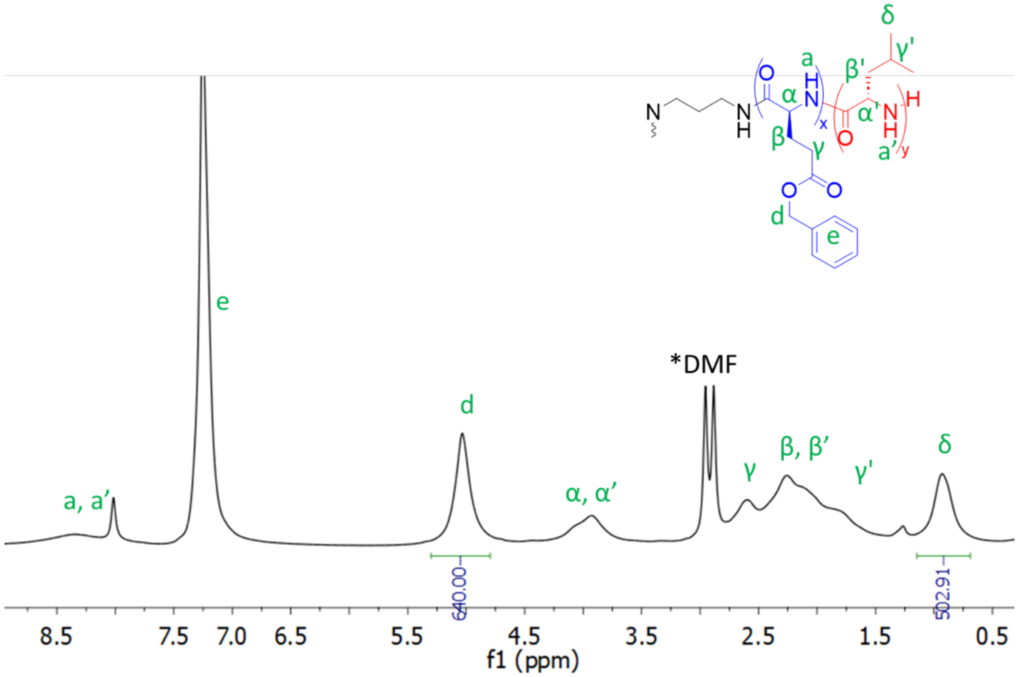

Figure S13. ^{1}H NMR spectra of 16-P(BLG$_{320}$-*b*-LLeu$_{80}$) in CDCl$_3$.

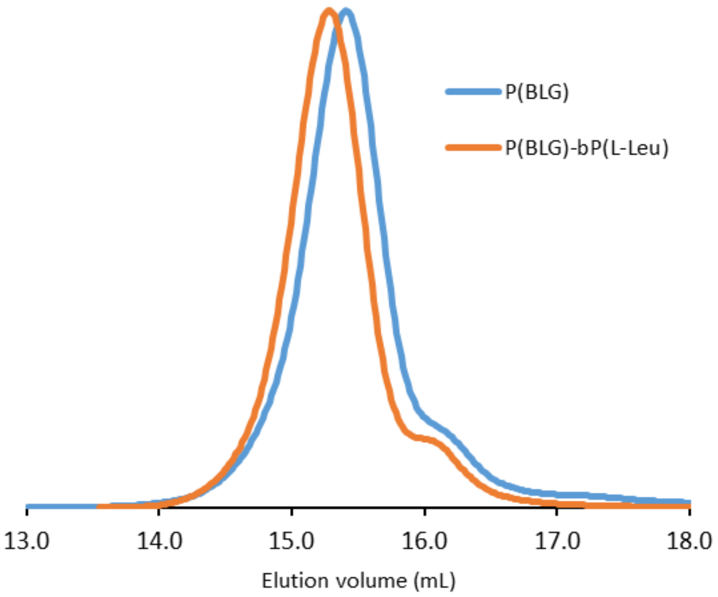

Figure S14. GPC trace illustrating peptide chain extension of 16-P(BLG$_{320}$) to 16-P(BLG$_{320}$-*b*-LLeu$_{80}$).

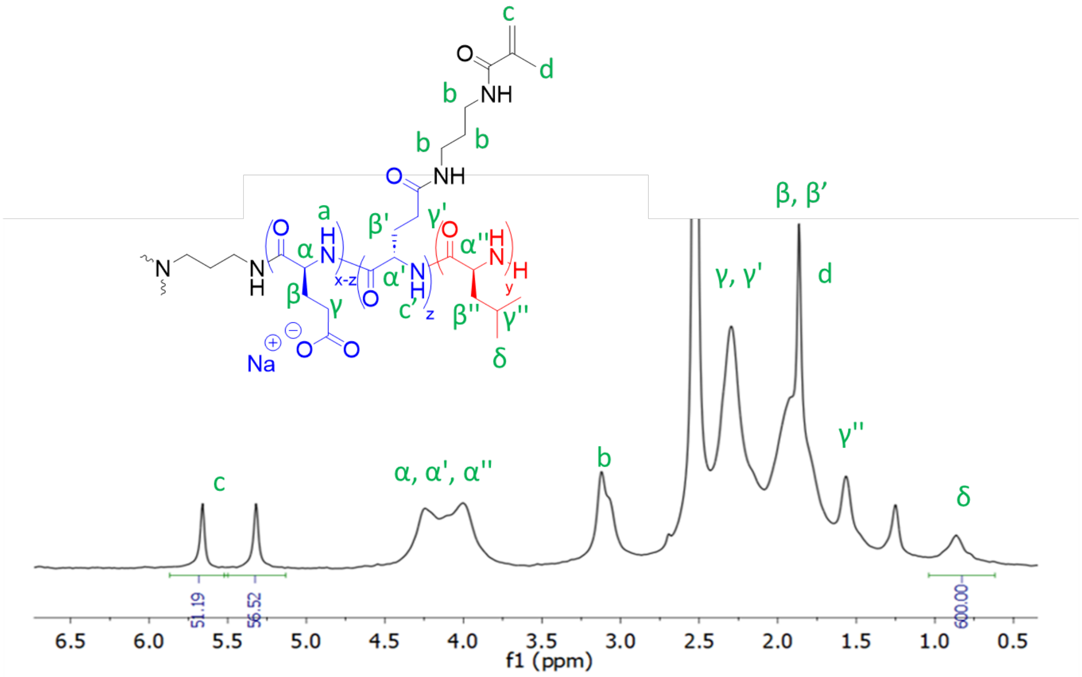

Figure S15. ^{1}H NMR spectra of methacrylamide functionalised glutamate units (target 20% of 320 units) of 4-SDC in DMSO-d_6/d-TFA as the solvent.

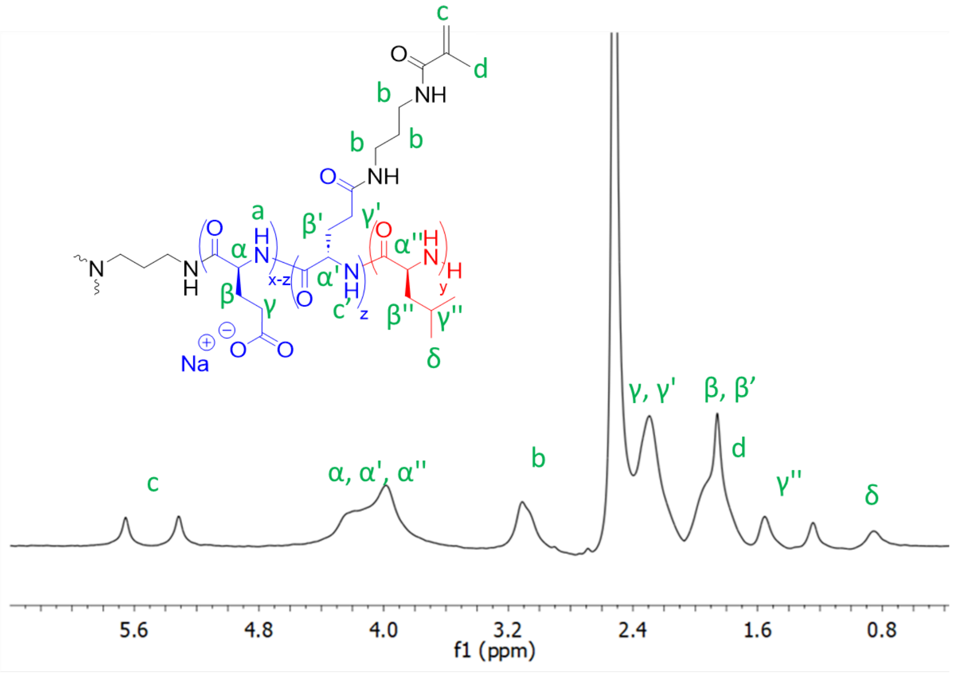

Figure S16. ^{1}H NMR spectra of methacrylamide functionalised glutamate units (target 20% of 320 units) of 8-SDC in DMSO-d_6/d-TFA as the solvent.

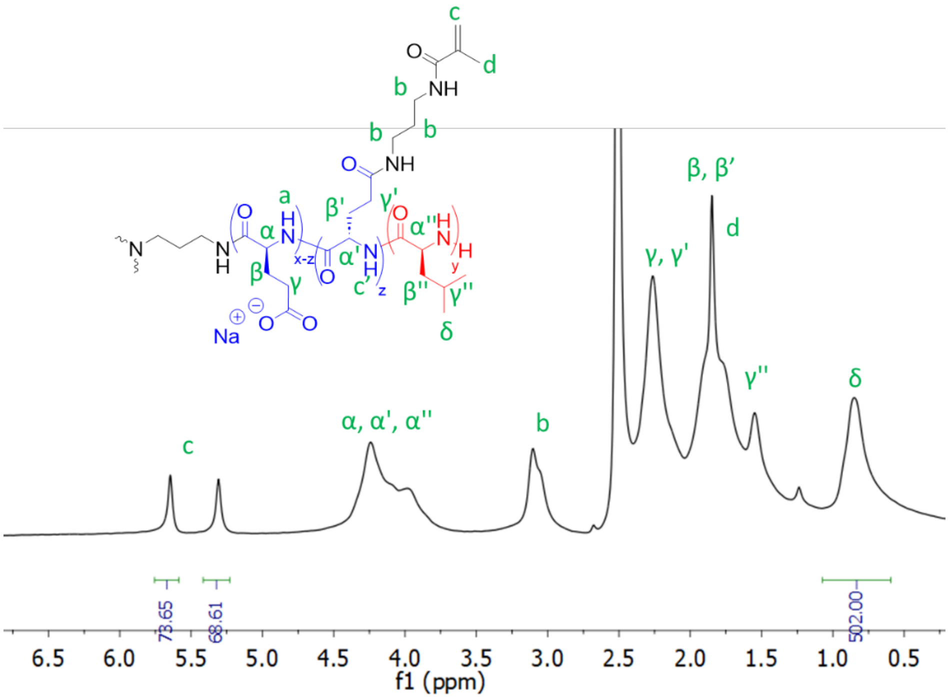

Figure S17. ^{1}H NMR spectra of methacrylamide functionalised glutamate units (target 20% of 320 units) of 16-SDC in DMSO-d_6/d-TFA as the solvent.

Table S2. Determination of star polypeptide secondary structures from circular dichroism spectra.

Polymer	***Helix***	***Sheet***	***Turn***	***Other***
4-SDC	72.5	2.0	2.6	22.9
8-SDC	73.5	16.6	9.8	0.1
16-SDC	21.1	23.6	15.2	40.1

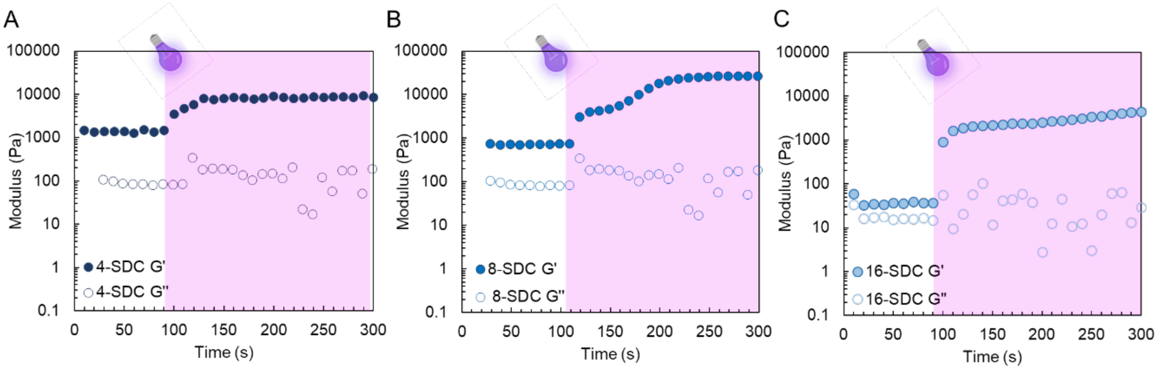

Figure S18. Rheological time curing sweep, showing photo-crosslinking of hydrogel comprising 5.0 wt% 4-SDC (A), 8-SDC (B) and 16-SDC (C) in 0.1 wt% LAP solution and 5.0 wt% acrylamide comonomer. Irradiation began at 90 seconds and was continuous using 405 nm LED (6 mW/cm^2), all experiments were performed at $\gamma = 0.1\%$ and $\omega = 1$ rad/s.

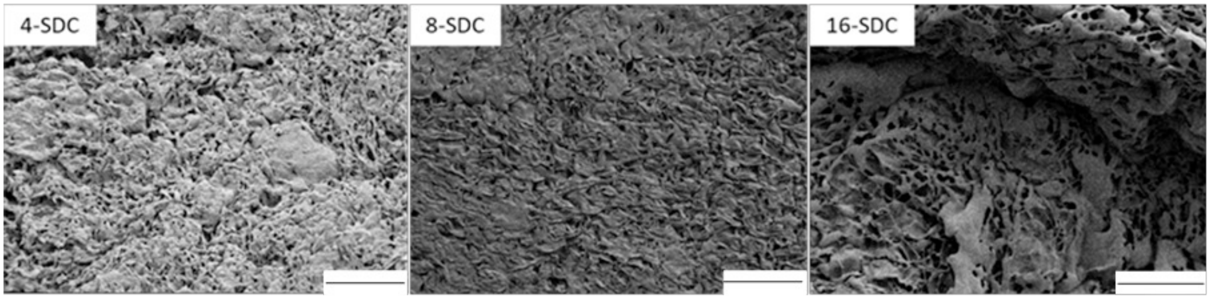

Figure S19. Morphological analysis of 4-SDC, 8-SDC, and 16-SDC by SEM (scale bar: 40 μm).
